# Supplementary figures and images for: Spatial regulation of Lck activation at the CD8 immune synapse revealed by a FRET-Based biosensor
Source: Cell Mol Life Sci. 2026 Apr 23;83(1):234. doi: 10.1007/s00018-026-06209-x (PMC13237420; doi:10.1007/s00018-026-06209-x)

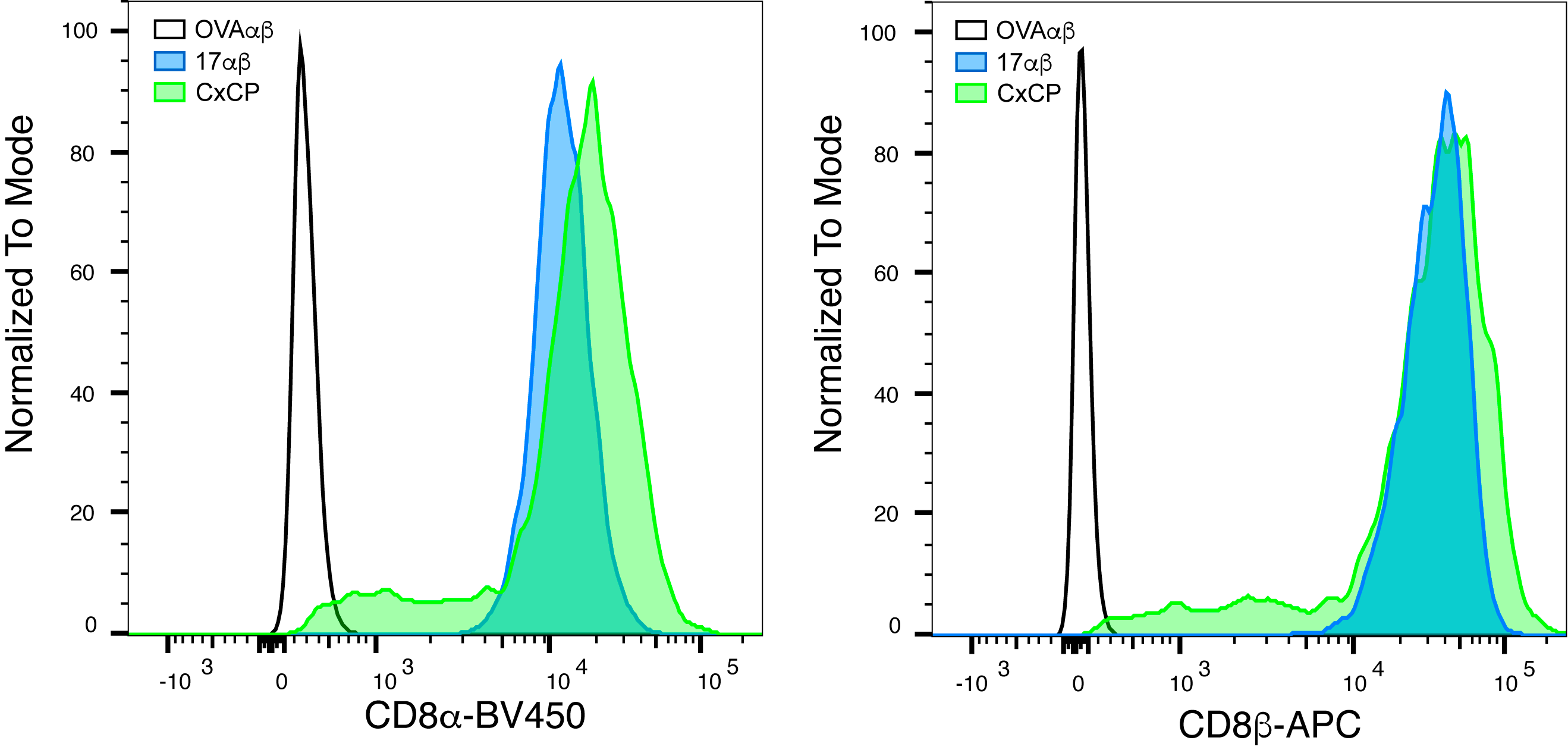

Supplement: Supplementary file 2 — Supplementary Material 1 (PNG) [file 18_2026_6209_Fig7_ESM.png]

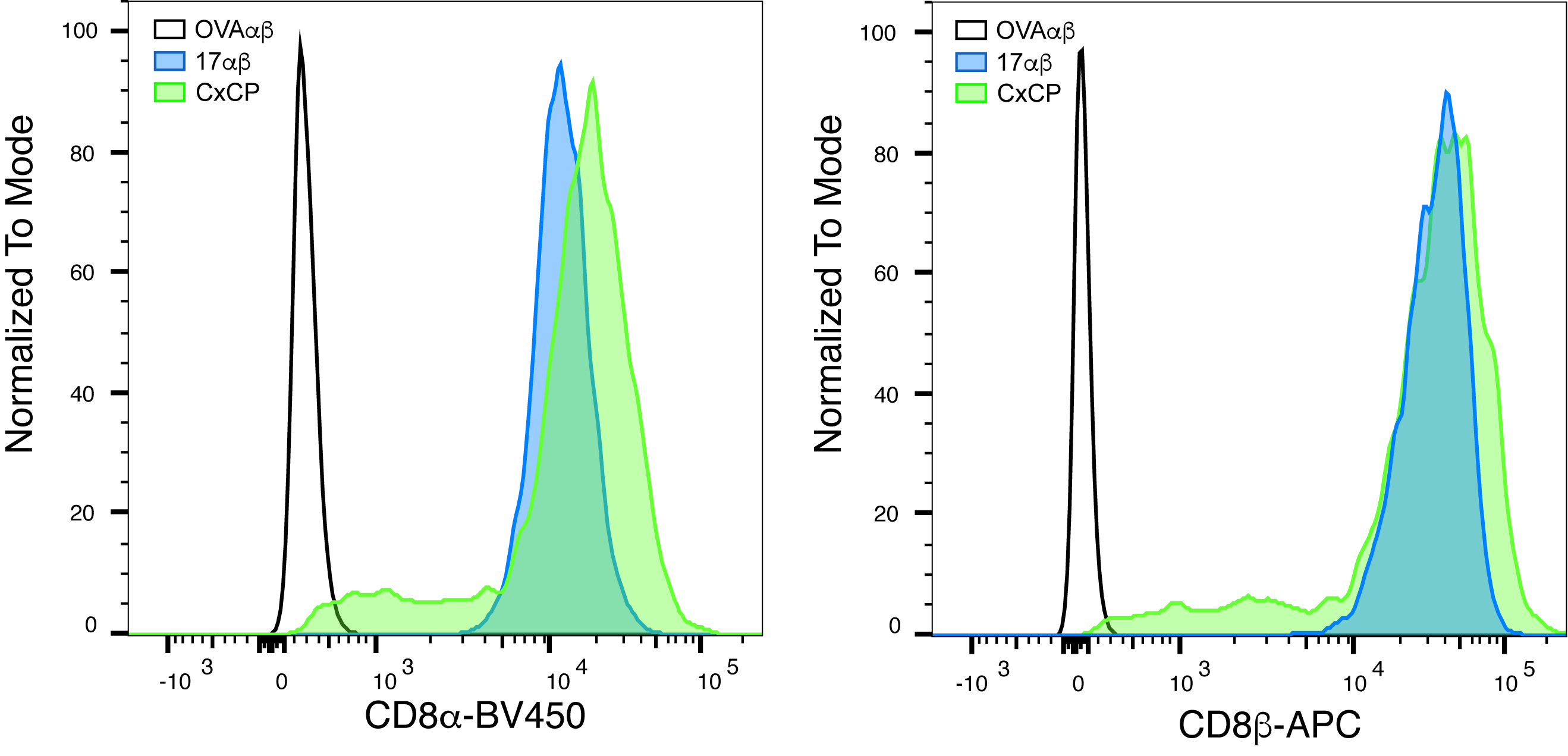

Supplement: Supplementary file 3 — High Resolution Image (TIF) [file 18_2026_6209_MOESM2_ESM.tif]

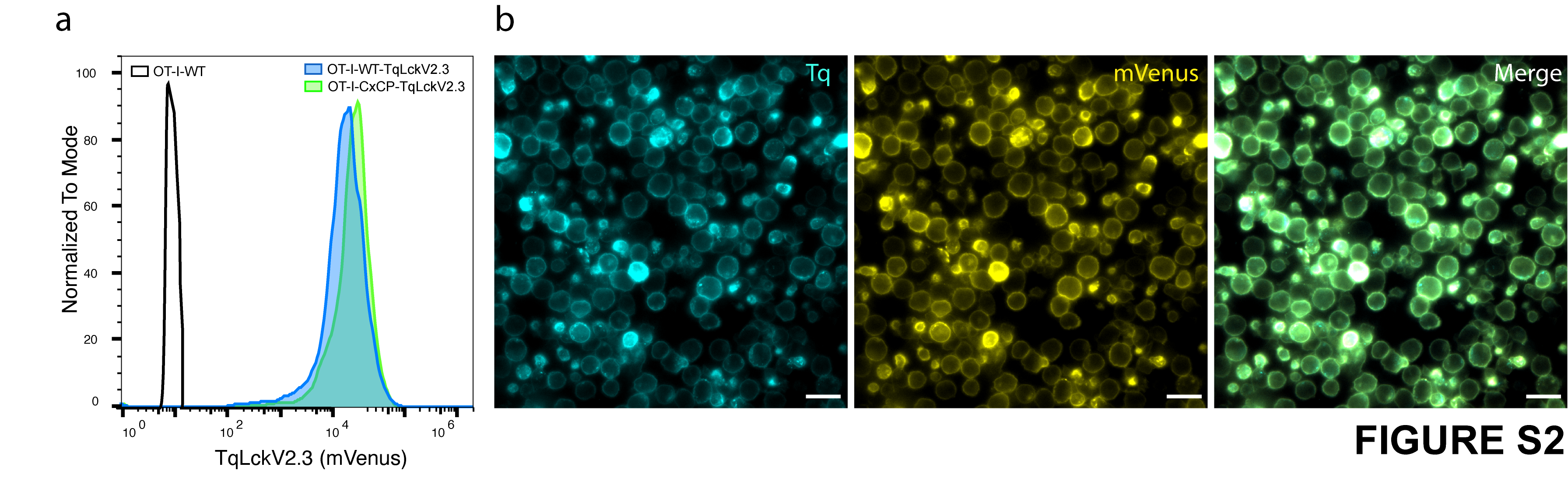

Supplement: Supplementary file 4 — Supplementary Material 2 (PNG) [file 18_2026_6209_Fig8_ESM.png]

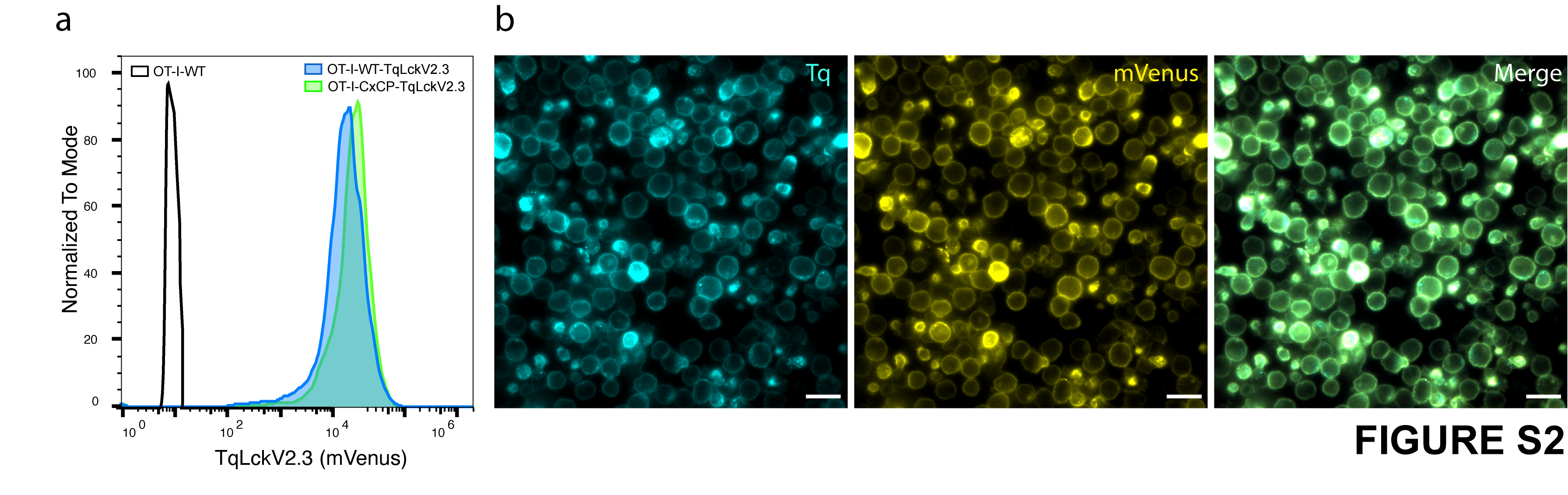

Supplement: Supplementary file 5 — High Resolution Image (TIF) [file 18_2026_6209_MOESM3_ESM.tif]

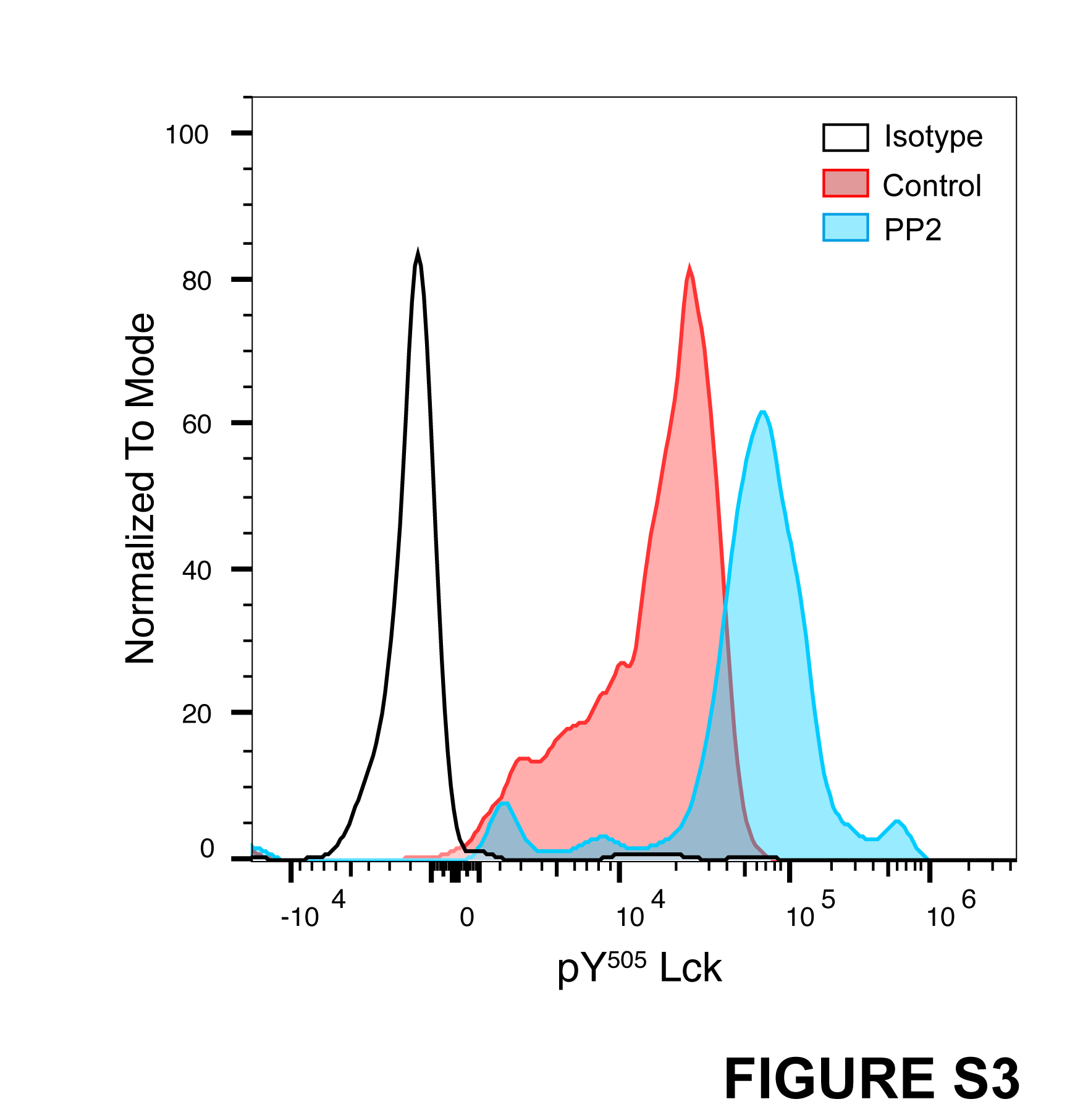

Supplement: Supplementary file 6 — Supplementary Material 3 (PNG) [file 18_2026_6209_Fig9_ESM.png]

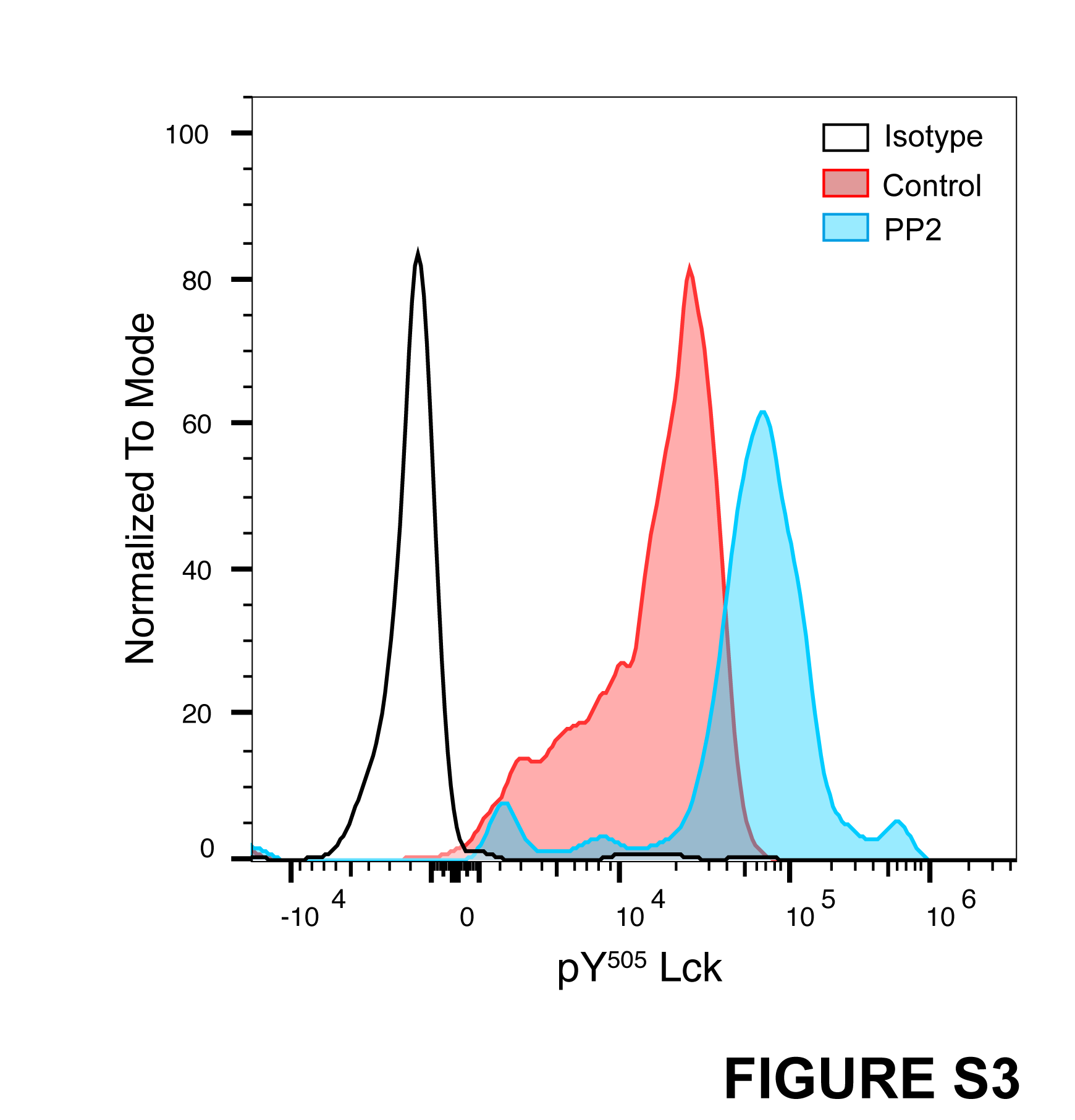

Supplement: Supplementary file 7 — High Resolution Image (TIF) [file 18_2026_6209_MOESM4_ESM.tif]

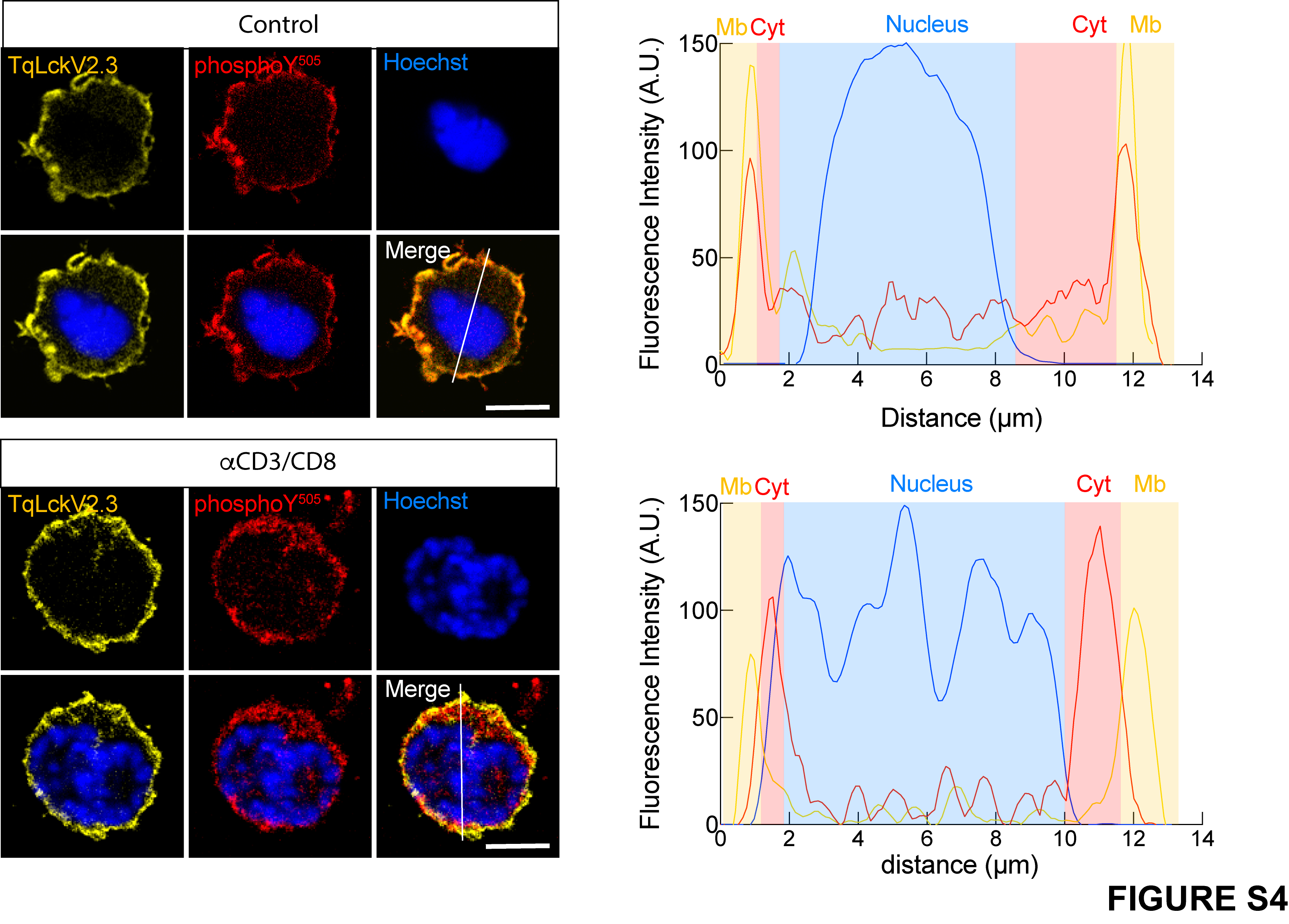

Supplement: Supplementary file 8 — Supplementary Material 4 (PNG) [file 18_2026_6209_Fig10_ESM.png]

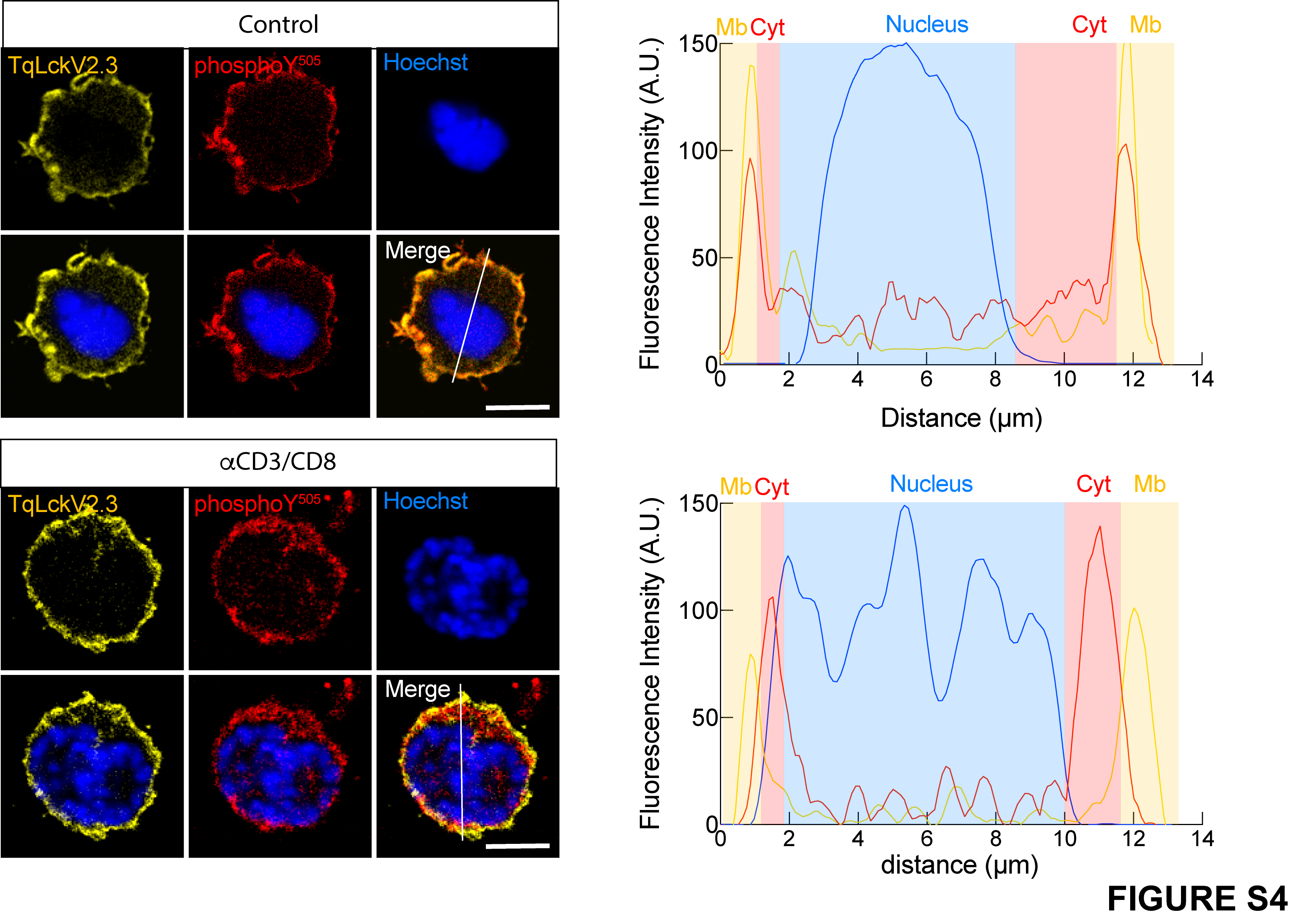

Supplement: Supplementary file 9 — High Resolution Image (TIF) [file 18_2026_6209_MOESM5_ESM.tif]
